# Casein/Apricot Filler in the Production of Flame-Retardant Polyurethane Composites

**DOI:** 10.3390/ma14133620

**Published:** 2021-06-29

**Authors:** Sylwia Członka, Agnė Kairytė, Karolina Miedzińska, Anna Strąkowska

**Affiliations:** 1 Institute of Polymer & Dye Technology, Lodz University of Technology, 90-924 Lodz, Poland; karolina.miedzinska@dokt.p.lodz.pl (K.M.); anna.strakowska@p.lodz.pl (A.S.); 2 Laboratory of Thermal Insulating Materials and Acoustics, Faculty of Civil Engineering, Institute of Building Materials, Vilnius Gediminas Technical University, Linkmenu St. 28, 08217 Vilnius, Lithuania; agne.kairyte@vgtu.lt

**Keywords:** polyurethanes, composites, flame retardancy, mechanical characteristic, thermal conductivity

## Abstract

Polyurethane (PUR) composites reinforced with 1, 2, and 5 wt.% of apricot filler modified with casein were synthesized in the following study. The impact of 1, 2, and 5 wt.% of casein/apricot filler on the cellular structure and physico-mechanical performances of reinforced PUR composites were determined. It was found that the incorporation of 1 and 2 wt.% of casein/apricot filler resulted in the production of PUR composites with improved selected physical, thermal, and mechanical properties, while the addition of 5 wt.% of casein/apricot filler led to some deterioration of their physico-mechanical performance. The best results were obtained for PUR composites reinforced with 2 wt.% of casein/apricot filler. Those composites were characterized by a uniform structure and a high content of closed cells. Compared with the reference foam, the incorporation of 2 wt.% of casein/apricot filler resulted in improvement in compressive strength, flexural strength, impact strength, and dynamic mechanical properties—such as glass transition temperature and storage modulus. Most importantly, PUR composites showed better fire resistance and thermal stability due to the good thermal performance of casein. The main aim of this article is to determine the influence of the natural combination of the apricot filler and casein on the mechanical properties and flammability of the obtained composites.

## 1. Introduction

Polyurethane (PUR) materials are widely used in many application, in the form of foams, resins, adhesives, coatings, elastomers, and sealants [1]. The wide range of applications is due to the variety of raw materials from which they are obtained: polyols and polyisocyanates with the addition of catalysts, blowing agents, flame retardants, and other modifiers [2]. Most of the basic ingredients of polyurethane are currently obtained from non-renewable petrochemical raw materials and may pose a risk to the environment [3,4]. Therefore, due to increasing environmental requirements, waste management issues, and the depletion of non-renewable resources, scientists are looking for new ecological solutions, such as the production of polyols from natural materials or the addition of natural waste as fillers [5,6,7,8].

One of the main groups of polyurethane materials are porous products—foams. In terms of their properties, they are divided into two main types: rigid polyurethane foams (RPUFs) and flexible polyurethane foams (FPUFs) [9,10]. PUR foams are mainly obtained by the one-step method. It is characterized by a two-component system in which the A component is a polyol system with catalysts, flame retardants, fillers, and other modifiers and the B component is an isocyanate system. During synthesis, two main processes take place: polymerization—leading to the formation of mainly urethane and urea bonds (the creation of a three-dimensional structure)—and foaming—the evaporation of the blowing agent, resulting in the expansion of the reaction mixture (the creation of a porous structure) [4]. Rigid polyurethane foams are characterized by a rigid, cross-linked and closed-cell structure. The properties of foams, such as low thermal conductivity and good dimensional stability, are determined by the cell structure of the obtained foams, especially by the closed-cell content. Higher closed-cell content limits heat transfer, which translates into better thermal-insulation properties. In terms of application, PUR foams are widely used in construction as thermal and acoustic insulation materials. Besides, they are used in many industries, including electronics, automotive, refrigeration, or furniture [11,12].

In recent years, there has been increasing interest in the use of natural and waste materials as fillers in polymer composites. The literature describes the use of nutshells, beet pulp, fruit seeds and pomace, linseed cake, or wheat straw as modifiers improving the properties of the obtained composite materials [13,14,15,16,17,18,19,20,21]. There are also biopolyols and isocyanates obtained from natural materials. The literature describes biopolyols that can be made of walnut shells, bamboo residues, cotton stalks, sugar cane, as well as cooking, rapeseed, sunflower, and soybean oils [4,18,22,23,24,25,26]. On the other hand, the selection of bio-based isocyanates is limited. The most common raw material for bio-based isocyanates are fatty acid derivatives [27]. Another noteworthy bio-based isocyanate is pentamethylene diisocyanate (PDI), which was the first bio-based diisocyanate to be commercialized [28]. Bio-based polyols and isocyanates are mainly used to reduce the proportion of petrochemical raw materials and increase the use of natural and renewable materials [29].

One reinforcing filler in the case of rigid polyurethane foams may be ground apricot (*Prunus armeniaca* L.) stones. Apricots belong to the *Rosaceae* family and occur both in domestic form (cultivated worldwide) and in a wild endemic form (Tian Shan Mountains, Central Asia) [30,31,32]. Apricot seeds were found in Garni (Armenia) in an archaeological excavation at the Chalcolithic-era site, hence its scientific name—*Prunus armeniaca* L. Apricots are mainly grown in Turkey, Uzbekistan, France, Iran, and Algeria. Apricot fruit can be eaten raw or dried, as well as in the form of jams or marmalades. Interestingly, apricot seed oil can be used in the pharmaceutical and perfumery industries [33]. Apricot stones can be a source of benzaldehyde. Additionally, apricot-seed essential oil has antibacterial and antimicrobial properties and can be used as an antimicrobial, disinfectant, and preservative agent [34,35]. Apricot stones mainly consist of lignin (47.97%), cellulose (29.57%), hemicelluloses (17.01%), and ash (0.95%) [36]. The density of the biofiller, which is the apricot stone, determined by Qaiss et al. [37], is 1.28 g cm^−3^. The same authors also performed FTIR and TGA experiments on the apricot filler and other fillers. Their research has shown that the use of natural fillers can effectively reinforce polymer composites. The high mechanical properties of the used fillers provide an increase in the mechanical properties of the obtained composites.

As for the use of polyurethane foams, the main disadvantage and limitation of their use are their fire behavior (easy ignition and high flammability). This significantly limits their use in many branches of engineering [38]. To increase the versatility of the application of polyurethane composite materials, research on reducing their flammability has begun. Various methods have been proposed to increase the fire resistance of obtained foams, including the formation of fire-retardant coatings on the foams surface, the application of compounds containing special, flame-retardant functional groups (especially hydroxyl groups), or the incorporation of anti-pyrenes into the reaction mixture [39]. In the past, flame retardants containing halogens were mainly used, but in recent years they have been banned due to their harm to the environment [40,41]. For this reason, halogen-free flame retardants have been applied as a replacement [42]. There are various types of non-halogen flame retardants, including expandable graphite, phosphorus, melamine compounds, and inorganic salts, but also inorganic metal (aluminum, silicon, or magnesium) oxides and hydroxides, as well as polyhedral oligomeric silsesquioxanes (POSS) [43,44,45,46,47,48,49].

**Figure 1:**
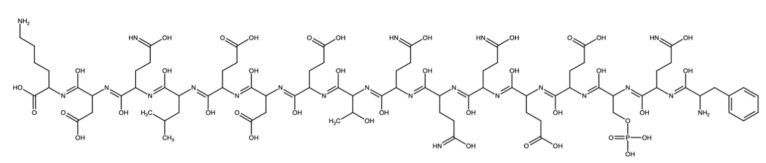
The chemical structure of casein.

One of the halogen-free, bio-based flame retardants is casein, which is a group of related phosphoproteins described with the chemical composition C_81_H_125_N_22_O_39_P. The high content of phosphorus and nitrogen favors the use of casein as a flame retardant for polymer materials. Its effectiveness has been confirmed so far on cotton and polyester fabrics and poly(lactic acid) composites [50,51,52]. The structure of casein is shown in Figure 1.

In the present study, the influence of the used modifiers on fire behavior and the mechanical properties of rigid polyurethane foams was determined. Despite many studies conducted on bio-fillers and their modifications that can be applied in polyurethane foams, to the best of our knowledge, there was no research on rigid polyurethane foams filled with apricot-stones filler modified with casein. The combination of apricot filler with casein results from the desire to simultaneously improve the mechanical properties and reduce the flammability of the obtained composites, assuming the use of raw materials of natural origin.

## 2. Materials and Methods

### 2.1. Methods

The viscosity of the polyol systems was determined using a Viscometer DVII+ (Brookfield, Germany) following ISO 2555. The apparent density was measured following the standard ASTM D1622, which is equivalent to ISO 845, as the ratio of sample weight to its volume. The density was measured on five samples of each foam and expressed as an average. Morphology and cell size distribution were examined based on the structure pictures of analyzed foams taken using JEOL JSM-5500 LV scanning electron microscopy (JEOL LTD, Akishima, Japan). The microscopic analysis was carried out in a high-vacuum mode and at an accelerating voltage of 10 kV. Foam samples were scanned in a parallel direction to the foam growth. The average cell-diameter and pore-size distribution were measured using ImageJ software (Media Cybernetics Inc., Rockville, MD, USA). A three-point bending test was conducted following the standard ASTM D7264, which is equivalent to ISO 178. Foam samples were bent with a speed of 2 mm min^−1^. For each foam series, at least five measurements were made. The obtained flexural stress at the break results for each sample was expressed as a mean value and averaged. The compressive strength was determined by the standard ASTM D1621, which is equivalent to ISO 844. The compressive strength (σ_10%_) of polyurethane foams was evaluated according to the ISO 844 standard. All samples were measured in the perpendicular and parallel direction to the foam growth direction using Zwick Z100 Testing Machine (Zwick/Roell Group, Ulm, Germany). The measurement was performed up to 10% of sample deformation (load cell of 2 kN, constant speed of 2 mm min^−1^). The flexural strength of polyurethane foams was evaluated according to the ISO 178 standard. The measurement was performed using Zwick Z100 Testing Machine (Zwick/Roell Group, Ulm, Germany) at a constant speed of 2 mm min^−1^. The impact examination was carried out following the standard ASTM D4812 on the pendulum 0.4 kg hammer impact velocity at 2.9 m s^−1^. The impact was measured at room temperature, 25 °C, on at least five samples of each type of foam with dimensions of 10 mm × 10 mm × 100 mm using Zwick Z100 Testing Machine (Zwick/Roell Group, Ulm, Germany). Dynamic mechanical analysis (DMA) was examined using an ARES Rheometer (TA Instrumentfs, New Castle, DE, USA). The thermal stability of foams was analyzed using a Mettler Toledo Thermogravimetric Analyzer TGA/DSC1 (Mettler Toledo, Greifensee, Switzerland). The experiment included analysis of the mass change as a function of temperature during thermal decomposition of the PU foams. The flame-retardant properties and burning behavior were analyzed using a cone calorimeter following the standard ISO 5660 in S.Z.T.K. TAPS (Maciej Kowalski Company, Saugus, Poland). The measurement for each foam was repeated on three samples and averaged. Fourier-transform infrared spectroscopy (FTIR) absorbance spectra were investigated within the 4000–400 cm^−1^ range (64 scans). The study was performed with the use of a Thermo Scientific Nicolet 6700 FTIR spectrometer (Thermo Fisher Scientific, Waltham, MA, USA) equipped with diamond Smart Orbit ATR sampling accessory. The size of particles in polyol dispersion (0.04 g L^−1^) was determined with the dynamic light scattering DLS method using a Zetasizer instrument (Malvern Instruments Ltd., Malvern, UK) with a 0.5 cm path length quartz cuvette. Samples were irradiated with red light (HeNe laser, wavelength λ = 632.8 nm) and the intensity fluctuations of the scattered light (detected at a backscattering angle of 173°) analyzed to obtain an autocorrelation function. The software (DTS v5.03) provided both the size mean and polydispersity, using the cumulants analysis (according to the international standard ISO 13321:1996) and a size distribution using a regularization scheme by intensity, volume, and number. Diffuse reflectance UV-Vis spectroscopy was performed on an Evolution 201/220 UV-Visible Spectrophotometer (Thermo Fisher Scientific, Waltham, MA, USA) with a 0.5 cm path length quartz cuvette. The absorption spectra of the filler solution (5 mg mL^−1^ in ethanol) were measured in the wavelength range from 200 to 700 nm at a 1 nm step with a scan speed of 8 nm s^−1^. The test was repeated three times for each sample. The thermal conductivity was determined using LaserComp 50 heat flow meter apparatus (TA Instruments, New Castle, DE, USA). The dimensional stability of foams was determined following the standard ASTM D2126, which is equivalent to ISO 2796. The dimensional stability under conditions of raised and lowered temperatures was determined based on the linear changes in dimensions, volume, and mass of PUR composites. Conditioning was carried out at −20 and +70 °C for 14 days. XRD experiment was performed using an X-ray diffractometer Bruker model D2 Phaser (Billerica, MA, USA), equipped with a Lynxeye detector (Billerica, MA, USA).

### 2.2. Materials

- Purocyn B (polymeric diphenylmethane diisocyanate) was purchased from Purinova Company (Bydgoszcz, Poland);
- Stepanpol PS-2352 (polyether polyol) was purchased from Stepan Company (Northfield, IL, USA);
- Catalysts: Kosmos 75 (potassium octoate) and Kosmos 33 (potassium acetate) were purchased from Evonik Industry (Essen, Germany);
- Surfactant: Tegostab B8513 (silicone-based surfactant) was purchased from Evonik Industry (Essen, Germany);
- Blowing agents: pentane and cyclopentane were purchased from Sigma-Aldrich Corporation (St. Louis, MO, USA);
- Casein powder (Sigma-Aldrich, St. Louis, MO, USA);
- Ground apricot stones were supplied by a local company (Lodz, Poland).

### 2.3. Synthesis of PUR Composites Reinforced with Casein/Apricot Filler

**Figure 2:**
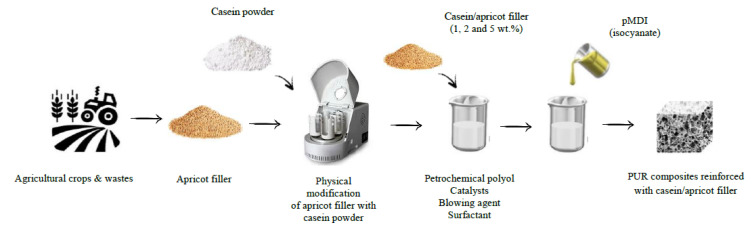
Schematic procedure of the synthesis of PUR composites reinforced with casein/apricot filler.

Before adding to the polyol system, apricot stone (AS) filler was mechanically ground with knife mill PULVERISETTE 11 (Fritsch, Idar-Oberstein, Germany) and sieved using a 100 µm sieve. In the next step, the apricot powder was mixed with casein powder (apricot weight to casein weight ratio = 1:2) and milled with a PULVERISETTE 5 Classic Line planetary ball mill (Fritsch, Idar-Oberstein, Germany) (60 min, 3000 rpm, ball weight to powder weight ratio = 12:1). Apricot filler modified with casein (AS_C) was used as the reinforcing filler in the synthesis of PUR composites. The impact of filler content on the selected physical and mechanical properties of PUR composites was determined. For this purpose, PUR composites were prepared with the addition of 1, 2, and 5 wt.% of apricot–casein filler. Each component of the PUR system was added based on the weight percentage of polyol (Stepanpol PS2352). The preweighed amount of polyol, blowing agent (pentane/cyclopentane), surfactant (Tegostab B8513), catalysts (Kosmos 33, Kosmos 75), and water were placed in the plastic container (volume of 1100 mL) and mechanically stirred for 60 s (2000 RPM). In the next step, the casein/apricot filler was added to the mixture and continuously stirred for another 60 s. After that, the polymeric diphenylmethane diisocyanate (Purocyn B) was added to the mixture, and the obtained system was mixed vigorously for 30 s (2000 RPM). As synthesized PUR composites were freely expanded and cured for 24 h at room conditions (temperature of ~22 °C, humidity of ~40%). The produced PUR composites were labeled PUR_REF, PUR_AS_C_1, PUR_AS_C_2, an PUR_AS_C_5, wherein the first one was the reference foam (without the addition of a filler), and the number represents the weight percentage of casein/apricot filler in final PUR composites. The formulations of PUR composites reinforced with casein/apricot filler are given in Table 1. The schematic procedure of the synthesis of PUR composites is represented in Figure 2.

## 3. Results and Discussion

### 3.1. Casein/Apricot Filler Characterization

#### 3.1.1. External Morphology of the Filler

**Figure 3:**
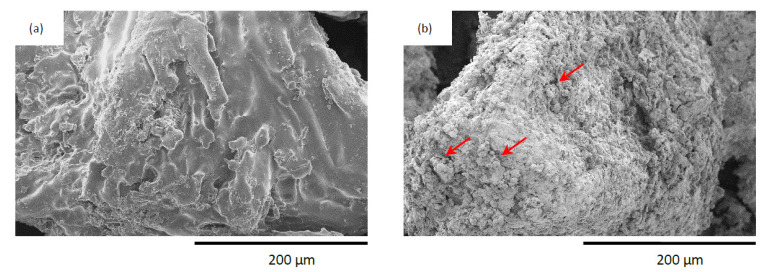
External morphology of (**a**) apricot filler, and (**b**) casein/apricot filler.

An external morphology of apricot filler and apricot filler modified with casein was investigated by scanning electron microscopy (SEM). The obtained images (Figure 3) revealed that, before the treatment, the surface of the apricot filler was quite smooth and uniform. After the physical modification of apricot filler with casein compounds, the overall structure of the filler became rougher, with visible casein particles located on the surface of the filler. It is believed that such developed filler will provide good interfacial interlocking between the filler surface and the PUR matrix, improving their compatibility during PUR synthesis, as well as ensuring the effective incorporation of the filler into the structure of the PUR composites.

#### 3.1.2. Size of Filler Particles

**Figure 4:**
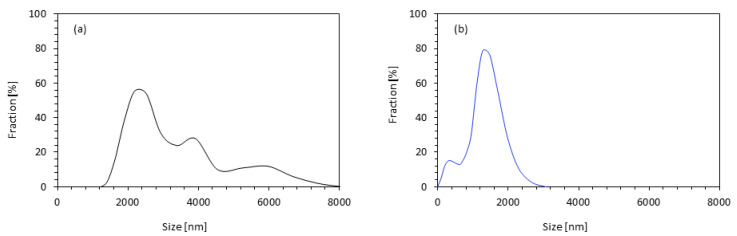
Particle-size distribution of (**a**) apricot filler and (**b**) casein/apricot filler.

According to the results of the dynamic light scattering (DLS) method (Figure 4a), most of the apricot filler particles are in the range of 2.5–3.0 µm; however, larger aggregates of apricot filler with an average size of ~4.0 µm were also observed in the PUR system. According to the results presented in Figure 4b, the casein/apricot mixtures mostly have 75% of their particles with an average size of 1.2–1.7 μm, 10% of their particles with a size of 0.5–1.2 μm, and 15% of their particles bigger than 1.2 µm. Thus, it can be concluded that the application of a high-energy ball-milling process results in the formation of a filler with a more homogenous particle-size distribution.

#### 3.1.3. Chemical Structure of the Filler

**Figure 5:**
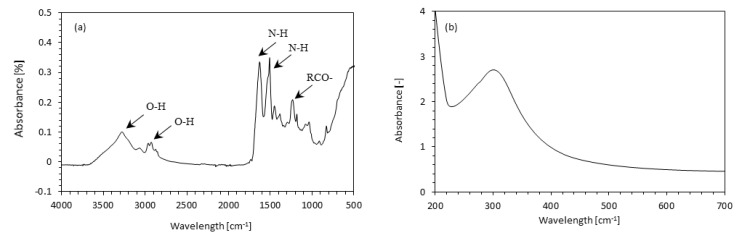
(**a**) FTIR spectrum and the (**b**) UV-Vis spectrum of casein/apricot filler.

The chemical structure of the casein/apricot filler was determined using FTIR and UV-Vis analysis. The obtained spectra are presented in Figure 5a,b, respectively. The FTIR spectrum of the casein/apricot filler confirms the presence of reactive groups in the filler molecule. The intense bands in the range of 1480, 1520, and 1610 cm^−1^ correspond to the vibration of -COO groups, N-H bending vibration (of secondary amines), and the N–H bending vibration (of primary amines) of protein molecules, respectively [53]. The presence of the bands located in the range of 3200–3500 cm^−1^ and 2900–3000 cm^−1^ confirms the presence of -OH groups in side chains and terminal groups [53]. The band located at 1210 cm^−1^ corresponds to the stretching vibration of acyl groups of casein molecules [54]. The UV-Vis spectrum of casein/apricot fillers exhibits an intense peak in the range of 250–310 nm. This confirms the presence of the basic amino acids tryptophan and tyrosine, which absorb UV light at a wavelength of 280–390 nm [55]. Other minor amino acids, such as glutamine and aspartic acid, absorb UV light at a wavelength of 295–305 nm.

### 3.2. Characterization of PUR Composites

#### 3.2.1. Analysis of the Foaming Characteristic and Dynamic Viscosity of PUR Systems Containing Casein/Apricot Filler

**Figure 6:**
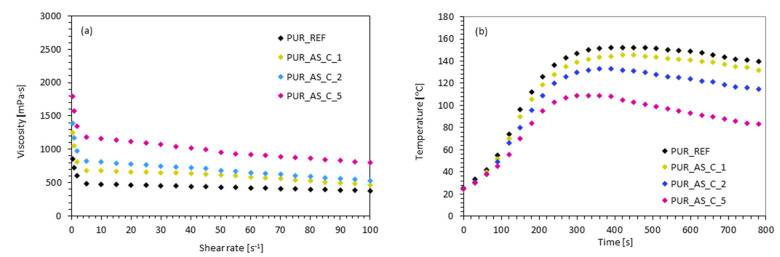
(**a**) Dynamic viscosity of PUR systems modified with casein/apricot filler and (**b**) maximum temperature measured during the synthesis of PUR composites.

The foaming kinetics of PUR composites reinforced with casein/apricot filler were evaluated by measuring the characteristic processing times—start, growth, and tack-free time. The results presented in Table 2 indicate that, with the increasing of the casein/apricot filler content, the processing times of modified PUR systems increased proportionally. With the addition of 1, 2, and 5 wt.% of casein/apricot filler the value of start time increased by ~15, ~27, and ~60%, respectively, while the value of growth time increased by ~8, ~13, and ~25%, respectively. The possible explanation of these extended times may be found in the dynamic-viscosity results. As presented in Figure 6a, the value of dynamic viscosity of modified PUR systems significantly increased from 810 ± 7 mPa·s (for PUR_REF) to 1250 ± 10, 1310 ± 8, and 1790 ± 10 mPa·s after the incorporation of 1, 2 and 5 wt.% of casein/apricot filler. This effectively disrupted the expansion process and further growth of the cells, increasing the start and growth time values. The effect is more evident after adding 5 wt.% of casein/apricot filler, leading to the conclusion that higher filler content leads to higher viscosity and consequently, longer processing times for PUR systems [56,57]. Additionally, extended processing times for PUR systems modified with casein/apricot filler may be related to the reduced amount of carbon dioxide (CO_2_) that is released from the reaction between the isocyanate (-NCO) and the hydroxyl (-OH) groups of the polyol. Some isocyanate groups are consumed in the reaction with functional groups (mostly amine groups, -NH_2_) of casein/apricot filler. This affects the proper stoichiometry of the PUR synthesis reaction, leading to the reduced amount of CO_2_ in the PUR systems. Given that CO_2_ acts as a chemical foaming agent in PUR systems, cell expansion is greatly reduced, and the growth time of the modified PUR systems is effectively extended. Confirmation may be found in the results of the maximum temperature measured during PUR synthesis (Figure 6b). With increases in the filler content, the maximum value of the maximum temperature decreased, which means that, with the addition of casein/apricot filler, the reactivity of the modified PUR systems was reduced [56,57]. Previous studies have reported that some heat released during the PUR synthesis may be absorbed by natural filler [58]. Such an explanation may also be found in the current study.

#### 3.2.2. Cellular Structure, Apparent Density, and Thermal Conductivity of PUR Composites Reinforced with Casein/Apricot Filler

**Figure 7:**
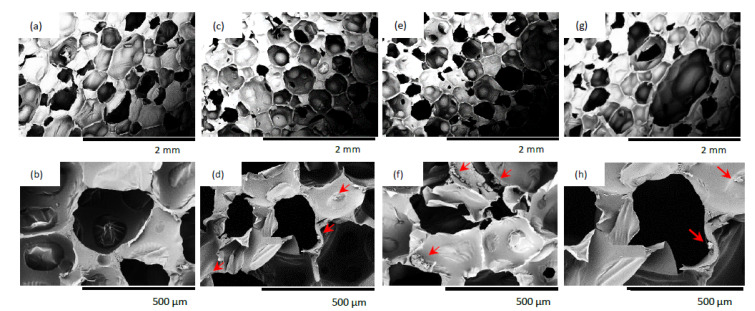
Cellular morphology of PUR composites: (**a**,**b**) PUR_REF; (**c**,**d**) PUR_AS_C_1; (**e**,**f**) PUR_AS_C_2; (**g**,**h**) PUR_AS_C_5.

The structure of the obtained PUR composites was analyzed using SEM images. The reference foam exhibited a relatively regular, hexagonal pore shape. As can be seen in Figure 7, the incorporation of apricot-stone filler modified with casein influences the morphology of the obtained PUR composites. As the filler content increased, the cellular structure of PUR composites became less homogeneous. A higher casein/apricot filler content also resulted in the formation of more open cells (i.e., the closed-cell content decreased). Additionally, filler particles can be observed in the cells. They may be built into the porous structure of the foam or remain inside the pores, which can result in the deterioration of the PUR structure and later mechanical properties.

**Figure 8:**
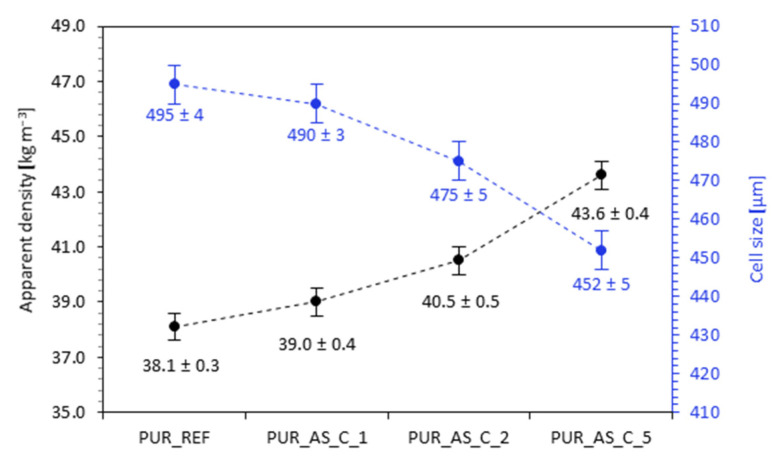
Apparent density and average cell diameter of PUR composites reinforced with casein/apricot filler.

**Figure 9:**
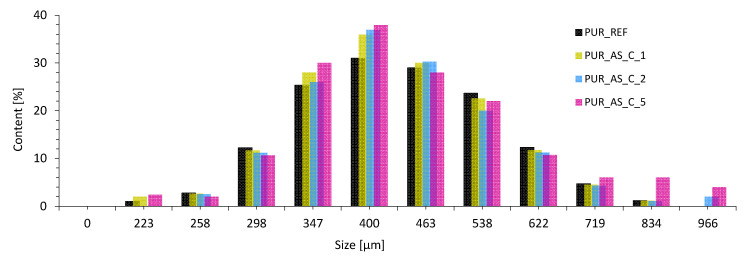
Percentage distribution of cell size in the structure of PUR composites.

As shown in Figure 8, the addition of casein/apricot filler influenced the apparent density and average cell diameter. The value of the apparent density increased from 38.1 kg m^−3^ for PUR_REF to 39.0, 40.5, and even 43.6 kg m^−3^ for PUR_AS_C1, PUR_AS_C2, and PUR_AS_C_5, respectively. Comparing the results of the average cell diameter with the reference foam, it can be seen that, in the case of modified foams, the average cell diameter decreased with increasing filler amount. On the other hand, based on the results shown in Figure 9 and Table 3, it is possible to notice some bimodality in the cell size of the porous structure. The average cell diameter is decreasing; however, there is an increase in the error range of this value and an increase in the number of both smaller and larger cells.

**Figure 10:**
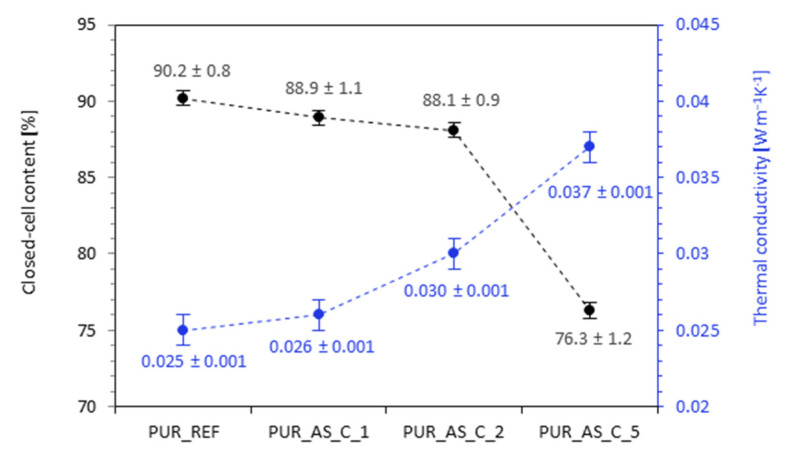
The results of closed-cells content and thermal-conductivity analysis.

When analyzing the data in Figure 10, it can be seen that the application of casein/apricot filler also had an impact on the content of closed cells and thus thermal conductivity. It is worth mentioning that thermal conductivity is one of the most important features of polyurethane foams when it comes to their use as thermal insulation materials in construction [59]. Comparing with the PUR_REF, in the case of PUR composites with the addition of casein/apricot filler, the closed-cells content decreased from 90.2% for PUR_REF to 88.9, 88.1, and 76.3% for PUR_AS_C_1, PUR_AS_C_2, and PUR_AS_C_5, respectively. When analyzing the thermal conductivity results, it can be seen that with the increase in the casein/apricot filler content, the thermal conductivity increases from the value of 0.025 W m^−1^ K^−1^ to 0.026, 0.030, and 0.037 W m^−1^ K^−1^ for PUR_AS_C_1, PUR_AS_C_2, and PUR_AS_C_5, respectively. The obtained results confirm the dependence of thermal conductivity on the content of closed cells, such that, with a decrease in the content of closed cells, the thermal conductivity increased [60,61]. The obtained results ranged between 0.025 and 0.037 m^−1^ K^−1^ and are good results, comparable with the results of other insulation materials used in construction; all series of PUR composites are in line with commercial requirements for commercial thermal insulation boards (according to ASTM E170).

#### 3.2.3. X-ray Diffraction Analysis of PUR Composites Reinforced with Casein/Apricot Filler

**Figure 11:**
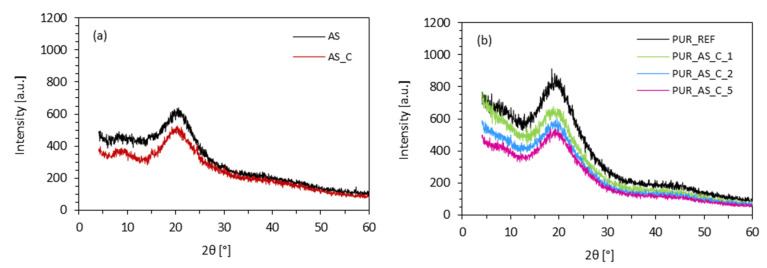
XRD patterns of (**a**) apricot-stone fillers and (**b**) PUR composites reinforced with casein/apricot filler.

X-ray diffraction (XRD) analysis was used to determine the effect of casein/apricot filler on the macro- and microstructure of PUR composites. Figure 11 shows the XRD patterns of apricot filler and casein/apricot filler. In both cases, an intense peak occurs at 2θ = 16–25°, which corresponds to the diffractions of amorphous cellulose I and amorphous cellulose II [49,50]. For the PUR_REF, wide diffraction from 15–25° with a maximum peak appeared at approximately 20°. For PUR composites, the addition of each amount of casein/apricot filler did not change the XRD patterns significantly. After the addition of 1 wt.% of casein/apricot filler, the intensity of the peak increased from 531 a.u. (for PUR_REF) to 545 a.u. On the further increasing the content up to 2 and 5 wt.% of the filler, the intensity decreased to 490 and 444 a.u., respectively. This may be related to the fact that the introduction of apricot-casein filler disturbed the originally uniform structure of PUR and caused PUR composites to become more amorphous. This effect is more prominent in the case of PUR composites containing 5 wt.% of casein/apricot filler, due to the greater number of open cells and more porous structure of such reinforced PUR composites. Thus, the intensity of the peak slightly decreased.

#### 3.2.4. Mechanical Properties of PUR Composites Reinforced with Casein/Apricot Filler—Compressive Strength, Flexural Strength, and Impact Strength

**Figure 12:**
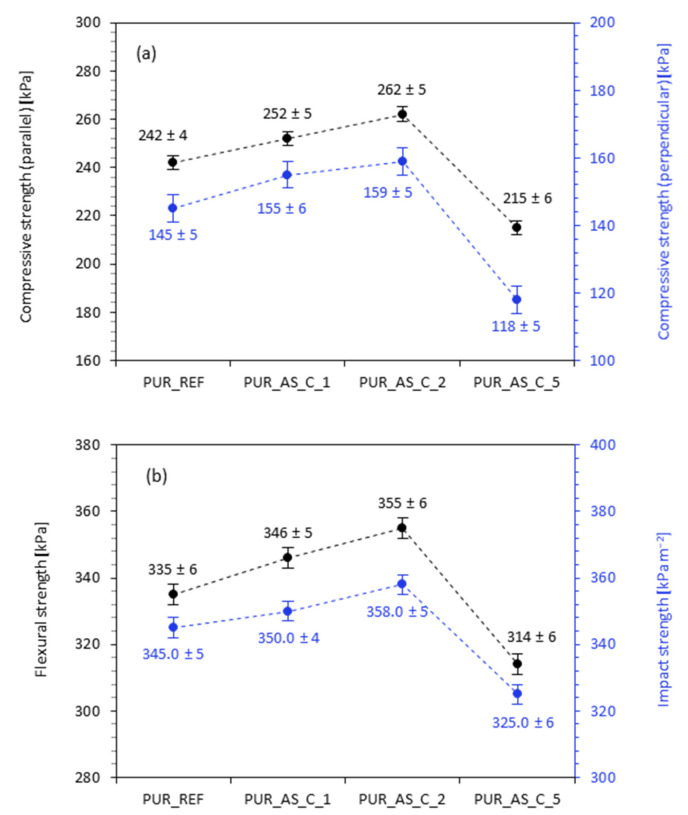
The mechanical performances of PUR composites reinforced with casein/apricot filler—(**a**) compressive strength and (**b**) flexural, impact strength.

The mechanical performances of PUR composites reinforced with casein/apricot filler were determined by performing compressive, flexural, and impact tests. The results of compressive strength—measured parallel and perpendicular to the foam growth direction—are presented in Figure 12a. According to the presented results, in both cases, the addition of 1 and 2 wt.% of casein/apricot filler results in the formation of PUR composites with improved mechanical strength. When compared to PUR_REF, the addition of 1 and 2 wt.% of casein/apricot filler increased the compressive strength (measured parallel) by ~5 and ~10%, respectively. An analog trend is observed in the case of compressive strength measured perpendicular to the foam direction—after the incorporation of 1 and 2 wt.% of casein/apricot filler, the value increased by ~7 and ~9%, respectively. In both cases, a significant deterioration in mechanical performances is observed after the addition of 5 wt.% of the filler—the value of compressive strength measured parallel and perpendicular decreased by ~11 and ~18%, respectively. The obtained results indicate that the addition of 1 and 2 wt.% of casein/apricot filler has a reinforcing effect, improving the mechanical performance of PUR composites. The incorporation of the casein/apricot filler in higher amounts, such as 5 wt.%, deteriorated the mechanical performance due to filler agglomeration and filler–filler interactions [62,63,64]. This in turn disrupted the foaming process, leading to the formation of PUR composites with a poor open-cell structure and deteriorated mechanical properties. To avoid the impact of apparent density on the compressive strength of modified PUR composites, the specific compressive strength (determined as a ratio of the compressive strength and apparent density of PUR composites) was calculated. The specific compressive strength of PUR_REF was 6.4 MPa kg^−1^m^−3^. After the addition of 1 and 2 wt.% of casein/apricot filler, the value increased to 6.5 MPa kg^−1^m^−3^ and then decreased to 4.9 MPa kg^−1^m^−3^, after the addition of 5 wt.% of the filler. Figure 12b presents the results of flexural and impact strength as a function of filler content. In the case of flexural strength, a significant improvement of this property is observed for PUR composites reinforced with 1 and 2 wt.%—when compared with PUR_REF, the value of flexural strength increased by ~3 and ~6%, respectively. This indicates an efficient filler–matrix interaction between the functional groups of casein/apricot filler and the PUR matrix, which may promote a strong anchorage, increasing the crosslinking density of PUR composites and their mechanical performances. The value of flexural strength decreased by ~6% as the amount of casein/apricot filler increased to 5 wt.%. This behavior has been observed in previous studies as well, and it is mainly connected with the poor structure of PUR composites and a greater number of broken cells. A very similar trend is observed in the case of impact strength results. Comparing to PUR_REF, the value of impact strength increased by ~2 and ~4% for PUR composites reinforced with 1 and 2 wt.% of the filler, due to filler–matrix adhesion, which results in load transfer between the matrix and filler and could be responsible for the additional improvement in the impact strength. The value of impact strength decreased by ~6% as the amount of the filler increased to 5 wt.%. This result may also be related to the fact that the filler particles act as additional stress points from which the composite cracking process begins [65,66]. As the concentration of casein/apricot filler increased over a certain optimal level, the effect was more prominent, leading to the deterioration of the abovementioned properties.

#### 3.2.5. Dynamic-Mechanical Analysis of PUR Composites Reinforced with Casein/Apricot Filler

**Figure 13:**
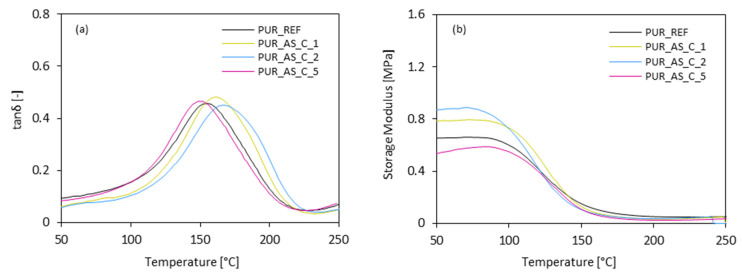
The results of dynamic mechanical analysis (**a**) tanδ and (**b**) storage modulus of PUR composites.

The results presented in Figure 13a indicate that the incorporation of casein/apricot filler affects the value of T_g_, which corresponds to the maximum value of the curve loss tangent (tanδ) versus temperature. When compared with PUR_REF, after the addition of 1 and 2 wt.% of casein/apricot filler, the values of T_g_ shifted towards higher temperatures. The highest value of T_g_ was exhibited in PUR composites reinforced with 2 wt.% of the filler—compared to PUR_REF, the value of T_g_ increased from 153 to 168 °C. These results are in agreement with the results of apparent density. As presented in Figure 8, the values of apparent density of PUR composites containing casein/apricot filler are somewhat higher, while the overall structure is quite uniform, with a high number of closed cells. This leads to the higher values of T_g_. The value of T_g_ decreased to 150 °C as the content of casein/apricot filler increased to 5 wt%. This effect may be connected with the poor interphase adhesion between the filler surface and the PUR matrix, which resulted in a more porous structure and contributed to an increase in the mobility of the polymer chains. The confirmation of the T_g_ results may be also found in the results of the storage modulus (E’). According to the results presented in Figure 13b, the incorporation of 1 and 2 wt.% of casein/apricot filler increased the value of E’. When compared with PUR_REF, the value of E’ increased by ~22 and ~35% after the addition of 1 and 2 wt.% of casein/apricot filler. This confirms the effective interactions between the casein/apricot filler and the PUR matrix, which are supported by the functional groups on the surface of the casein, thereby improving the reinforcement of the PUR composites. This indicates effective filler–matrix interaction due to the presence of isocyanate groups. The value of E’ decreased by 10% as the content of the casein/apricot filler increased to 5 wt.%, due to the more porous structure of PUR composites.

#### 3.2.6. Flame-Retardant Behavior of PUR Composites with Casein/Apricot Filler

The flame-retardant properties and burning behavior of PUR composites were assessed using the cone calorimetry method. Table 4 presents summarized results obtained during the experiment, including the ignition time (IT), the peak rate of heat release (pHRR), the total heat release (THR), the total smoke release (TSR), the average yield of CO and CO_2_ (COY and CO_2_Y), and the limiting oxygen index (LOI).

**Figure 14:**
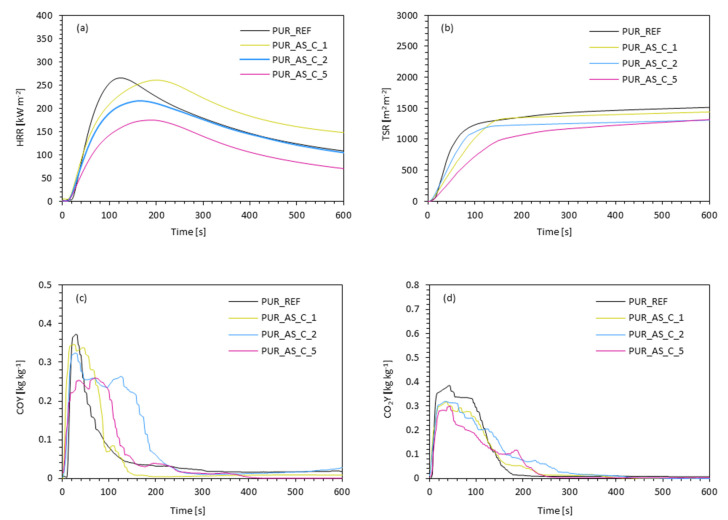
The results of the cone calorimeter test: (**a**) the peak rate of heat release (pHRR), (**b**) the total smoke release (TSR), (**c**) the average yield of CO, and (**d**) the average yield of CO_2_.

When analyzing the results obtained in Table 4, it can be seen that the filler addition influenced all tested parameters. Comparing the ignition time with the reference foam, an increase from 4 s for PUR_REF to 5 s for PUR_AS_C1 and PUR_AS_C2 and to 6 s for PUR_AS_C_5. The intensity of the flame is related to the release of low-molecular-weight compounds (amines, olefins, or isocyanates). This parameter was measured by the peak rate of heat release (pHRR). As Figure 14 presents, all samples show one peak of this indicator. Its values decreased from 266 kW m^−2^ for PUR_REF foam to 261, 216, and 175 kW m^−2^ respectively for PUR_AS_C_1, PUR_AS_C_2, and PUR_AS_C_5. When analyzing the influence of the apricot filler content on the total heat release (THR) and total smoke release (TSR) values, an analogous relationship is observed—the value of both parameters decreased compared to the reference foam (THR—21.7 MJ m^−2^, TSR—1650 m^2^ m^−2^), reaching the lowest value for PUR_AS_C_2 (THR—21.0 MJ m^−2^, TSR—1405 m^2^ m^−2^), and then increased for PUR_AS_C_5 (THR—21.5 MJ m^−2^, TSR—1590 m^2^ m^−2^).

**Figure 15:**
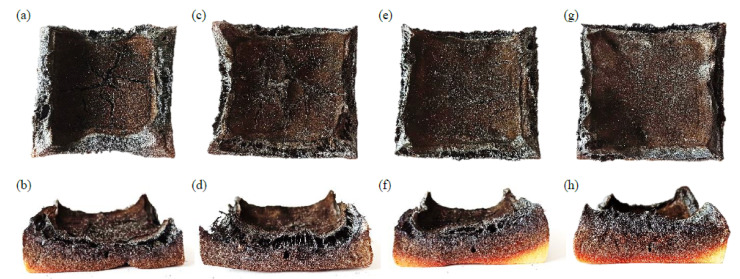
Polyurethane foams residues after the combustion process: (**a**,**b**) PUR_REF, (**c**,**d**) PUR_AS_C_1, (**e**,**f**) PUR_AS_C_2, and (**g**,**h**) PUR_AS_C_5.

The inclusion of apricot fillers also affected the gases released during combustion. Based on the data presented in Table 4, it can be seen that the incorporation of apricot fillers reduced the average yield of CO compared to the reference foam (0.373 kg kg^−1^). The values of this parameter were respectively 0.346 kg kg^−1^ for PUR_AS_C_1, 0.323 kg kg^−1^ for PUR_AS_C_2, and 0.254 kg kg^−1^ for PUR_AS_C_5. A similar trend is observed in the case of the average yield of CO_2_. Its values decreased from 0.385 kg kg^−1^ for the reference foam to 0.319, 0.318, and 0.298 kg kg^−1^ for PUR_AS_C_1, PUR_AS_C_2, and PUR_AS_C_5, respectively. Regarding the limiting oxygen index (LOI), it can be seen that the value of this index decreased with the increase in the apricot filler content. All the LOI values of the modified foams were greater than in the reference one (20.3%). LOI values reached 21.1, 21.8, and 22.5% for PUR_AS_C_1, PUR_AS_C_2, and PUR_AS_C_5, respectively. Based on the obtained results, it can be concluded that the incorporation of apricot filler modified with casein improves the flammability properties of the obtained PUR composites. Figure 15 presents samples of the analyzed PUR composites after the combustion process.

#### 3.2.7. Thermogravimetric Analysis (TGA) of PUR Composites Reinforced with Casein/Apricot Filler

**Figure 16:**
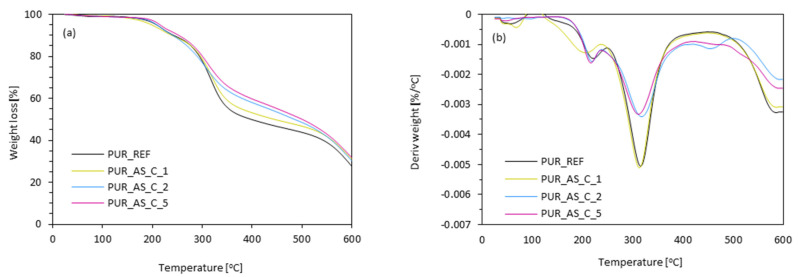
(**a**) Thermogravimetric (TGA) and (**b**) derivative thermogravimetry (DTG) results were obtained for PUR composites reinforced with casein/apricot filler.

To assess the effect of apricot filler modified with casein on the thermal stability of the PUR composites, thermogravimetric analysis (TGA) and derivative thermogravimetry analysis (DTG) were conducted. During the study, the stages of thermal decomposition were determined. Moreover, char residues at the temperature of 600 °C were assessed. The results obtained during the analysis are presented in Figure 16 and summarized in Table 5.

The thermal degradation of polyurethane foams is usually divided into three stages. The individual stages correspond to characteristic temperatures (T_max_) determined based on the results obtained during thermogravimetric analysis (TGA). The first stage of thermal degradation (T_max1_) is related to the thermal decomposition of low-molecular-weight compounds [67,68,69,70]. In the case of the analyzed PUR composites, the first stage of decomposition occurred between 205 and 221 °C. It can be seen that the incorporation of apricot filler resulted in higher values of T_max1_, which could be related to the modification of the filler with a natural flame retardant, which casein is [52]. The value of T_max1_ increased from 205 °C (PUR_REF) to 221, 219, and 217 °C for PUR_AS_C_1, PUR_AS_C_2, and PUR_AS_C_5, respectively. Thus, it can be seen that the greatest improvement at this stage is observed for the foam containing 1 wt.% of casein/apricot filler relative to polyol weight. The second stage of thermal degradation is the thermal degradation of hard segments of PUR composites and apricot filler [60,71,72]. It occurred in the temperature range between 313 and 317 °C. Based on the obtained results, it can be concluded that the influence of apricot filler in this stage of thermal degradation on the thermal stability of PUR composites was negligible. The last, third stage of thermal degradation is the degradation of the compounds and fragments that were generated during the previous stages [73]. In the case of the analyzed PUR composites, it occurred in the temperature range between 587 and 591 °C. The incorporation of casein/apricot filler resulted in a slight increase in the temperature characteristic for this stage T_max3_.

By analyzing the influence of the casein/apricot filler on the thermal stability of the obtained PUR composites, the char residue amount at 600 °C was also measured. When comparing the carbonization residue with the reference foam result, it can be seen that, with increasing apricot filler content, the residue content also increased. The content of char residue increased from 27.6% for PUR_REF to 30.3, 31.0, and 31.9%, respectively, for PUR_AS_C_1, PUR_AS_C_2, and PUR_AS_C_5. Based on the obtained results, it can be concluded that the application of apricot filler modified with casein increased the thermal stability of the obtained PUR composites and resulted in an increase in the content of residues after combustion at 600 °C.

#### 3.2.8. Dimensional Stability of the PUR Composites Reinforced with Casein/Apricot Filler

The dimensional stability under conditions of raised and lowered temperatures was determined based on the linear changes in dimensions, volume, and mass of the PUR composites. Conditioning was carried out at −20 and +70 °C for 14 days. The results of the analysis are presented in Table 6.

Compared to PUR_REF, the addition of casein/apricot filler resulted in slight changes in linear dimensions, volume, and mass. In the case of conditioning at low temperature (−20 °C), it can be seen that, in the case of modified PUR composites, the measured changes increased with increasing filler amounts. At this temperature, the best results were obtained for PUR_AS_C_1, which showed lower values in all parameters compared to PUR_REF. In the case of conditioning PUR composites at higher temperatures (+70 °C), the influence of the apricot filler on the obtained results can also be seen. The best results were observed for PUR_AS_C_1 (all parameter values were lower than for PUR_REF) and for PUR_AS_C_2 (more significant reduction of ∆l and ∆V, but greater value of ∆m than in case of PUR_REF), while in the case of PUR_AS_C_5, the deterioration of properties is observed (higher values of all measured changes compared to the reference foam). Following the construction standard, polyurethane composites used in construction should not show more than 3% changes in dimension linearity [74]. In the case of all of the obtained PUR composites, both the reference and those modified by the addition of apricot filler modified with casein, this condition was met.

## 4. Conclusions

Polyurethane (PUR) composites reinforced with 1, 2, and 5 wt.% of apricot filler modified with casein were synthesized in the following study. The impact of 1, 2, and 5 wt.% of casein/apricot filler on the cellular structure and physico-mechanical performances of reinforced PUR composites was determined. It was found that the incorporation of 1 and 2 wt.% of casein/apricot filler resulted in the production of PUR composites with improved selected physical, thermal, and mechanical properties, while the addition of 5 wt.% of casein/apricot filler led to some deterioration of their physico-mechanical performance. The best results were obtained for PUR composites reinforced with 2 wt.% of casein/apricot filler. Those composites were characterized by a uniform structure and a high content of closed cells. Compared with reference foam, the incorporation of 2 wt.% of casein/apricot filler resulted in a slight improvement of compressive strength (improvement by ~10%), flexural strength (improvement by ~9%), impact strength (improvement by ~5%), and dynamic mechanical properties—such as glass transition temperature and storage modulus. The glass transition temperature increased from 153 to 168 °C, while the storage modulus increased by ~35%. The PUR composites were characterized by better thermal stability, as well as improved flame retardancy—e.g., the peak rate of heat release (pHRR) decreased from 266 to 216 kW m^−2^; the total smoke release (TSR) decreased from 1650 to 1405 m^2^ m^−2^, while the limiting oxygen index (LOI) increased from 20.3 to 22.5%. The results presented in the current study confirmed that the addition of a proper amount of casein/apricot filler could be an effective, inexpensive, and environmentally friendly approach to the synthesis of PUR composites with improved physicomechanical, thermal, and fire-retardant performance.

## Figures and Tables

**Table 1:** Formulations of PUR composites prepared in the current study.

System	Compound	Content (wt.% to Polyol)
Polyol system	Stepanpol PS2352	100
	Pentane/cyclopentane	11
	Tegostab B8513	2.5
	Kosmos 33	6
	Kosmos 75	0.8
	Water	0.5
	Casein/apricot filler	0, 1, 2, 5
Isocyanate system	Purocyn B	160

**Table 2:** Processing times of PUR systems reinforced with casein/apricot filler.

Sample	Processing Times [s]		
	Start Time	Growth Time	Tack-Free Time
PUR_REF	39 ± 3	282 ± 9	360 ± 10
PUR_AS_C_1	45 ± 2	308 ± 8	345 ± 9
PUR_AS_C_2	49 ± 2	322 ± 6	350 ± 7
PUR_AS_C_5	55 ± 1	365 ± 7	355 ± 7

**Table 3:** Percentage distribution of cell size.

Size [µm]	Content [%]			
0–223	1.1 ± 0.3	2.1 ± 0.4	0.0 ± 0.0	1.5 ± 0.3
224–258	3.2 ± 0.4	3.3 ± 0.5	2.4 ± 0.3	2.1 ± 0.3
259–298	8.1 ± 0.4	7.4 ± 0.5	5.8 ± 0.4	5.4 ± 0.5
299–347	18.3 ± 0.5	18.5 ± 0.3	20.1 ± 0.6	22.4 ± 0.6
348–400	24.4 ± 0.6	25.4 ± 0.6	30.5 ± 0.6	28.6 ± 0.7
401–463	22.6 ± 0.6	21.6 ± 0.7	22.6 ± 0.7	21.4 ± 0.7
462–538	17.7 ± 0.5	14.4 ± 0.6	11.6 ± 0.5	9.4 ± 0.4
539–622	4.5 ± 0.3	7.5 ± 0.4	5.4 ± 0.4	5.7 ± 0.4
623–719	2.4 ± 0.4	2.1 ± 0.3	2.1 ± 0.5	3.1 ± 0.2
720–834	1.3 ± 0.3	1.4 ± 0.2	1.5 ± 0.4	2.1 ± 0.2
835–966	0.0 ± 0.0	0.0 ± 0.0	2.1 ± 0.3	2.2 ± 0.3

**Table 4:** The results of the cone calorimeter test.

Sample	IT (s)	pHRR (kW m^−2^)	THR (MJ m^−2^)	TSR (m^2^ m^−2^)	COY (kg kg^−1^)	CO_2_Y (kg kg^−1^)	LOI (%)
PUR_REF	4 ± 0	266 ± 7	21.7 ± 1.8	1650 ± 10	0.37 ± 0.01	0.38 ± 0.01	20.3 ± 0.4
PUR_AS_C_1	5 ± 0	261 ± 6	21.3 ± 1.7	1550 ± 12	0.34 ± 0.02	0.32 ± 0.02	21.1 ± 0.4
PUR_AS_C_2	5 ± 0	216 ± 6	21.0 ± 2.2	1405 ± 12	0.32 ± 0.01	0.32 ± 0.01	21.8 ± 0.3
PUR_AS_C_5	6 ± 0	175 ± 8	21.5 ± 2.4	1590 ± 14	0.25 ± 0.01	0.29 ± 0.01	22.5 ± 0.5

**Table 5:** The results of thermal stability of PUR composites.

Sample	T_max_ (°C)			Char Residue (wt.%) at 600 °C
	1st Stage	2nd Stage	3rd Stage	
PUR_REF	205 ± 4	313 ± 4	587 ± 4	27.6 ± 0.2
PUR_AS_C_1	221 ± 5	315 ± 5	587 ± 5	30.3 ± 0.1
PUR_AS_C_2	219 ± 2	317 ± 4	589 ± 5	31.0 ± 0.2
PUR_AS_C_5	217 ± 5	313 ± 4	591 ± 6	31.9 ± 0.2

**Table 6:** Changes in linear dimensions (∆l), volume (∆V), and mass (∆m) after conditioning at –20 and +70 °C, of PUR composites.

Sample	Temperature of −20 °C			Temperature of +70 °C		
	∆l (%)	∆V (%)	∆m (%)	∆l (%)	∆V (%)	∆m (%)
PUR_REF	1.79 ± 0.01	1.66 ± 0.01	1.77 ± 0.01	1.74 ± 0.01	1.43 ± 0.01	1.52 ± 0.01
PUR_AS_C_1	1.76 ± 0.01	1.64 ± 0.01	1.69 ± 0.01	1.68 ± 0.01	1.42 ± 0.01	1.49 ± 0.01
PUR_AS_C_2	1.78 ± 0.01	1.71 ± 0.01	1.78 ± 0.01	1.63 ± 0.01	1.40 ± 0.01	1.55 ± 0.01
PUR_AS_C_5	1.84 ± 0.01	1.79 ± 0.01	1.81 ± 0.01	1.79 ± 0.01	1.50 ± 0.01	1.61 ± 0.01

## Data Availability

Data sharing not applicable.
